# Derlin-3 Is Required for Changes in ERAD Complex Formation under ER Stress

**DOI:** 10.3390/ijms21176146

**Published:** 2020-08-26

**Authors:** Yuka Eura, Toshiyuki Miyata, Koichi Kokame

**Affiliations:** 1 Department of Molecular Pathogenesis, National Cerebral and Cardiovascular Center, Osaka 564-8565, Japan; eura@ncvc.go.jp (Y.E.); miyata@ncvc.go.jp (T.M.); 2 Department of Cerebrovascular Medicine, National Cerebral and Cardiovascular Center, Osaka 564-8565, Japan

**Keywords:** ER stress, ERAD, ERAD complex, Herp, Derlin-1, Derlin-2, Derlin-3, HRD1

## Abstract

Endoplasmic reticulum (ER)-associated protein degradation (ERAD) is a quality control system that induces the degradation of ER terminally misfolded proteins. The ERAD system consists of complexes of multiple ER membrane-associated and luminal proteins that function cooperatively. We aimed to reveal the role of Derlin-3 in the ERAD system using the liver, pancreas, and kidney obtained from different mouse genotypes. We performed coimmunoprecipitation and sucrose density gradient centrifugation to unravel the dynamic nature of ERAD complexes. We observed that Derlin-3 is exclusively expressed in the pancreas, and its deficiency leads to the destabilization of Herp and accumulation of ERAD substrates. Under normal conditions, Complex-1a predominantly contains Herp, Derlin-2, HRD1, and SEL1L, and under ER stress, Complex-1b contains Herp, Derlin-3 (instead of Derlin-2), HRD1, and SEL1L. Complex-2 is upregulated under ER stress and contains Derlin-1, Derlin-2, p97, and VIMP. Derlin-3 deficiency suppresses the transition of Derlin-2 from Complex-1a to Complex-2 under ER stress. In the pancreas, Derlin-3 deficiency blocks Derlin-2 transition. In conclusion, the composition of ERAD complexes is tissue-specific and changes in response to ER stress in a Derlin-3-dependent manner. Derlin-3 may play a key role in changing ERAD complex compositions to overcome ER stress.

## 1. Introduction

The accumulation of misfolded proteins in the endoplasmic reticulum (ER) can result in a cellular condition called ER stress. ER stress is associated with various diseases, such as neurodegenerative diseases, metabolic diseases, inflammatory diseases, and cancer [1]. To overcome this stress, the cell upregulates a variety of proteins transcriptionally and translationally [2,3]. Some of these are involved in a specialized protein disposal system called ER-associated protein degradation (ERAD). ERAD is an intracellular quality control system for eliminating unfolded, misfolded, and orphan proteins, including glycoproteins (ERAD substrates) from the ER. The process begins with the recognition of ERAD substrates, which are then transported to the cytosol, ubiquitinated, and degraded by the proteasome [4,5,6].

In mammals, ERAD substrates are recognized through the concerted functions of multiple proteins, including GRP78, calnexin, calreticulin, ER mannosidase I, EDEM, ERdj5, and OS-9 [7,8,9,10,11,12]. Once recognized, ERAD substrates are transferred to retrotranslocation channels and secreted into the cytosol. During or after retrotranslocation, ERAD substrates are ubiquitinated by ubiquitin-protein E3 ligases, such as HRD1 and gp78, and thereby targeted to the 26S proteasome [13,14,15]. Concomitantly, glycoprotein substrates are deglycosylated by a cytosolic peptide, N-glycanase [16].

In the ER membrane, proteins involved in the ERAD system (ERAD factors) form ERAD complexes that perform the abovementioned functions of the ERAD system on substrates that carry lesions either on ER luminal domains (ERAD-L), membrane domains (ERAD-M), or cytosolic domains (ERAD-C) [4,5,6]. HRD1, a central player in the ERAD-L system, forms a high-molecular mass complex with SEL1L, Herp, Derlin-1, p97, and others [17,18,19]. HRD1, Derlin-1, and Sec61 have been considered potential components of the retrotranslocon [20,21,22,23]. Recent studies based on cryogenic electron microscopy [24,25] and electrophysiological analysis [26] revealed that yeast HRD1 forms a retrotranslocation pore. In mammals, the Derlin family consists of three members: Derlin-1, Derlin-2, and Derlin-3. Among these, Derlin-2 and Derlin-3 are involved in the degradation of glycosylated ERAD substrates [27], and they are redundant for the degradation of SEL1L-dependent substrates such as ATF6 [28].

The physiological significance of Derlin-3 is not well-investigated. Our previous data showed that Derlin-3-deficient mice were viable and appeared normal [29]. Derlin-3 deficiency, however, reduced the expression levels of Derlin-1 and Derlin-2 in the pancreas but not in the liver and kidney. We also found that the expression of Derlin-3 was induced by ER stress in the mouse liver and kidney but not in the pancreas [29]. The pathological study demonstrated that Derlin-3 is the most inducible protein downstream of the transcription factor ATF6 in the ischemic heart and contributes to the protection of cardiomyocytes against ischemic stress [30]. Derlin-3 is involved in colon cancer through the Warburg effect [31] and in breast cancer through the promotion of malignant phenotypes [32]. It was also reported that Derlin-3 inhibits the malignant evolution of gastric cancer and may be associated with immunomodulatory functions [33]. Considering these findings, we sought to explore the molecular function of Derlin-3 in vivo.

In our previous study, we also developed Herp-deficient mice, which exhibit a vulnerability to ER stress and defects in the degradation of ERAD substrates in the liver [29]. Herp-deficient mice also had defects in cardiac function and the insulin signaling in skeletal muscle [34,35]. Herp is highly induced by ER stress [36,37] and plays a role in the regulation of the HRD1-mediated ubiquitination of ERAD substrates [38].

In the present study, we investigated the significance of Derlin-3 through the biochemical characterization of the ERAD complexes present in the ER membrane of mouse organs. We reveal that at least three types of ERAD complexes exist, and their compositions differ in response to ER stress in a Derlin-3-dependent manner. Derlin-3 may exert a key role in changing the ERAD complex composition to overcome ER stress.

## 2. Results and Discussion

### 2.1. Expression of Herp and Derlins in Mouse Organs

**Figure 1:**
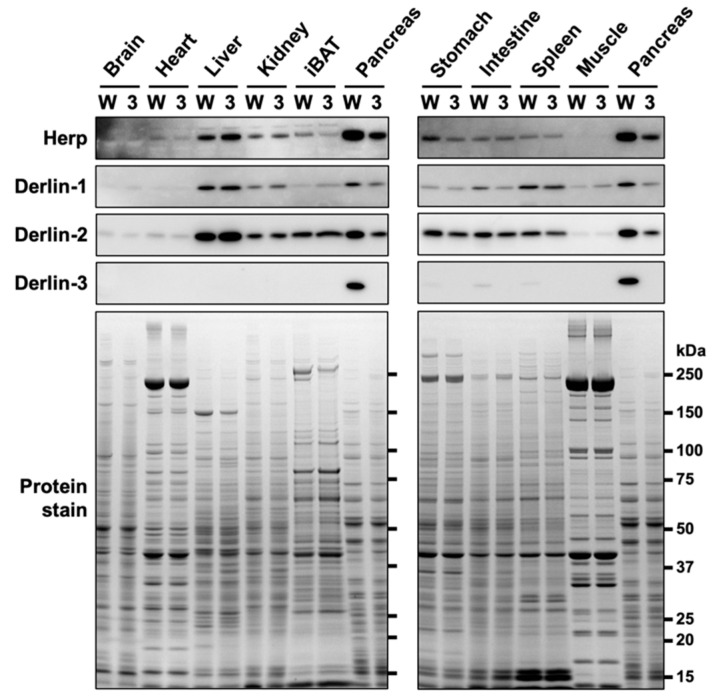
Protein expression patterns in mouse organs. Wild-type (WT) (W) and Derlin-3-deficient (3KO) (3) mice were fasted for 12 h before euthanasia, and organ homogenates were subjected to a Western blotting analysis using antibodies indicated on the left side. Bottom images (Protein stain) show protein bands in the gel stained with GelCode Blue Stain Reagent to check for equal loading. iBAT, interscapular brown adipose tissue.

In our previous study [29], we showed that Derlin-3 was constitutively expressed in the adult mouse pancreas. However, in the liver and kidney, Derlin-3 expression was detected only under ER stress conditions. In the present study, we analyzed the expression of Herp and Derlins in ten organs of wild-type (WT) and Derlin-3-deficient (3KO) mice (Figure 1). Derlin-3 showed highly restricted expression in the pancreas and low expression in the digestive organs and spleen. Derlin-1, Derlin-2, and Herp displayed ubiquitous expressions, with a wide range of expression levels among the analyzed organs. These observations are consistent with the mRNA expression data in the Human Protein Atlas (www.proteinatlas.org). As reported previously [29], the expressions of Derlin-1 and Derlin-2 were decreased in the 3KO pancreas, as compared to that in the WT. We refer to this observation again later, in Section 2.3.

In contrast to the findings of our previous study [29], the protein level of Herp was clearly reduced in the pancreas of 3KO mice (Figure 1). We noted that the discrepancy in the Herp level between our previous and present findings may be because of the difference in the time taken to prepare organ homogenates (three organs from each mouse in the previous study, and ten organs from each mouse in the present study). Herp may be preferentially degraded during sample preparation in the absence of Derlin-3. To test this hypothesis, we investigated the stability of Herp in the homogenates of the pancreas.

### 2.2. Herp Is Rapidly Degraded in the Derlin-3-Deficient Pancreas

**Figure 2:**
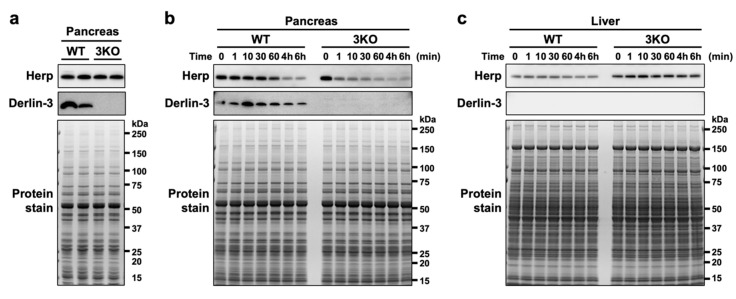
Rapid degradation of Herp in the Derlin-3-deficient pancreas. WT and 3KO mice were fasted for 12 h before euthanasia. Pancreas (**a**,**b**) and liver (**c**) homogenates in SDS sample buffer (**a**) or phosphate-buffered saline (PBS) (**b**,**c**) were incubated on ice for the indicated times (0–60 min, 4 h, and 6 h). The homogenates were subjected to a Western blotting analysis using antibodies indicated on the left side. Bottom images (Protein stain) show protein bands in the gel stained with GelCode Blue Stain Reagent to check for equal loading.

To observe the protein stability of Herp in the pancreas, we analyzed the Herp levels in pancreas homogenates incubated on ice at various time points. When the pancreatic tissues were homogenized in the denaturing, SDS-containing sample buffer just after isolation, the Herp levels were comparable between the WT and 3KO pancreas (Figure 2a); this was consistent with our observations from the previous study [29]. Incubation of the pancreatic homogenates in nondenaturing phosphate-buffered saline (PBS) on ice demonstrated a gradual decrease in Herp levels in WT and a rapid decrease in 3KO (Figure 2b), suggesting that Herp was readily degraded in the absence of Derlin-3. On the contrary, the levels of Herp were stable in the WT and 3KO liver homogenates, where the expression of Derlin-3 was not detected (Figure 2c). From these results, we concluded that Herp was rapidly degraded in the absence of Derlin-3 in organs in which Derlin-3 is normally expressed, suggesting that Derlin-3 can affect the molecular surroundings of Herp.

### 2.3. Derlin-3 Contributes to the Stability of Derlin-1 and Derlin-2 in a Herp-Dependent Manner

**Figure 3:**
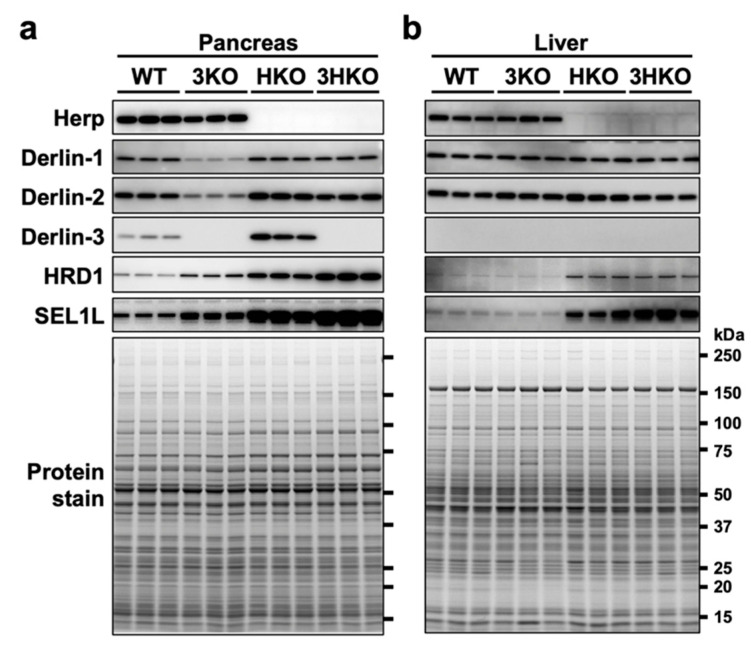
The effect of Derlin-3 and Herp deficiency on the protein expression levels of ER-associated protein degradation (ERAD) factors. WT, 3KO, Herp-deficient (HKO), and Derlin-3/Herp double-deficient (3HKO) mice (*n* = 3) were fasted for 12 h before euthanasia. Pancreas (**a**) and liver (**b**) homogenates (each mouse per lane) were subjected to Western blotting using antibodies indicated on the left side. Bottom images (Protein stain) show protein bands in the gel stained with GelCode Blue Stain Reagent to check for equal loading.

The protein levels of Derlin-1 and Derlin-2 were also reduced in the 3KO pancreas (Figure 1). To investigate the protein vulnerability caused by Derlin-3 deficiency in the pancreas, we generated Derlin-3/Herp double-deficient (3HKO) mice by the interbreeding of 3KO [29] and Herp-deficient (HKO) mice [29] and compared the protein levels of Derlin-1 and Derlin-2 among WT, 3KO, HKO, and 3HKO mice. In the pancreas, the protein levels of Derlin-1 and Derlin-2 were decreased in 3KO mice, as compared to those in the WT; however, this was not the case in HKO and 3HKO mice (Figure 3a). This showed that the decrease in Derlin-1 and Derlin-2 levels in the 3KO pancreas was dependent on the presence of Herp. Herp deficiency itself did not show any effect on the Derlin-1 and Derlin-2 levels. Derlin-3 may stabilize Derlin-1 and Derlin-2 in the presence of Herp, and in the absence of Herp, Derlin-3 was not required to maintain the Derlin-1 and Derlin-2 levels. However, Herp deficiency resulted in the increase in Derlin-3 levels (Figure 3a, HKO). These findings may suggest that Herp and Derlin-3 potentially have a functional interaction.

### 2.4. Herp and Derlin-3 Are Cooperatively Engaged in the Degradation of HRD1 and SEL1L

In the pancreas, the levels of HRD1 and SEL1L were elevated because of Herp deficiency (Figure 3a, HKO), which is consistent with the findings of the previous reports stating that HRD1 is degraded by the 26S proteasome in a Herp-dependent manner [38,39,40] and that SEL1L forms a complex with HRD1 [40]. The elevation of HRD1 and SEL1L levels in the HKO pancreas was further enhanced by Derlin-3 deficiency (Figure 3a, 3HKO), suggesting that the proteasomal degradation of HRD1 and SEL1L was dependent on the presence of Derlin-3. These findings suggested that Herp and Derlin-3 in the pancreas are cooperatively engaged in the degradation of HRD1 and SEL1L. These effects of Derlin-3 gene disruption in the pancreas were not observed in the liver, where the expression of Derlin-3 was not detected (Figure 3b).

### 2.5. Physical Interactions among ERAD Factors—Complex-1a and Complex-1b

**Figure 4:**
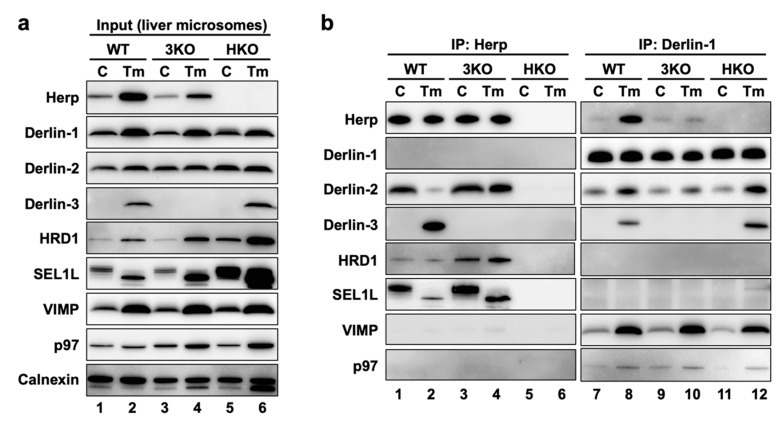
ERAD complexes in the mouse liver with or without ER stress. WT, 3KO, and HKO mice were intraperitoneally injected with PBS as control (C) or tunicamycin (Tm) (2-µg/g body weight) 24 h before euthanasia; the mice were fasted for 12 h before euthanasia. Livers were homogenized and subjected to subcellular fractionation using centrifugation. Digitonin-solubilized liver microsomes were prepared, and coimmunoprecipitation was performed using anti-Herp and anti-Derlin-1 antibodies. The input (**a**) and eluates (**b**) were analyzed by Western blotting using antibodies indicated on the left side. The loading volumes of the samples were adjusted to equalize the band intensities of the directly immunoprecipitated target proteins (Herp and Derlin-1).

Next, we sought to investigate the interactions among ERAD factors, including Derlins and Herp. The mouse liver is a suitable organ to biochemically investigate the effects of Derlin-3 expression, because its expression was not detected under normal conditions (Figure 1, Figure 2 and Figure 3) and dramatically increased at both the mRNA and protein levels under the ER stress condition induced by the peritoneal injection of tunicamycin (Tm) [29]. The highly regulated expression of Derlin-3 suggested that the presence or absence of Derlin-3 may be important for the function of ERAD. To test the idea, we investigated the molecular behavior of ERAD factors in the mouse liver. The livers were excised from WT, 3KO, and HKO mice that had been treated with PBS (control, C) or Tm for 24 h, and the microsome fractions were collected. The microsomes were solubilized with 1% digitonin and subjected to the coimmunoprecipitation experiments, followed by a Western blotting analysis (Figure 4).

**Figure 5:**
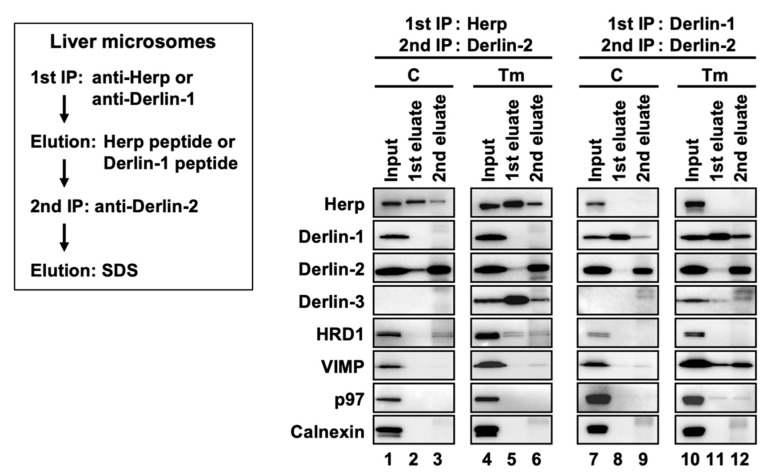
Sequential coimmunoprecipitation of ERAD factors. WT mice were injected intraperitoneally with PBS (C) or Tm 24 h before euthanasia. Digitonin-solubilized liver microsomes were prepared and subjected to sequential coimmunoprecipitation using anti-Herp (first) and anti-Derlin-2 (second) antibodies (left panels) or anti-Derlin-1 (first) and anti-Derlin-2 (second) antibodies (right panels). The eluates were analyzed by Western blotting using antibodies indicated on the left side. IP, immunoprecipitation.

First, we confirmed that the expression of the ERAD factors was induced under ER stress in the WT mice livers (Figure 4a, lanes 1 and 2). Due to the inhibition of protein N-glycosylation by Tm, the electrophoretic mobility of SEL1L (a glycoprotein) was enhanced, and the corresponding (nonglycosylated) band was localized slightly lower than the original (glycosylated) band. In the coimmunoprecipitation experiments, anti-Herp antibodies precipitated Herp, along with Derlin-2, HRD1, and SEL1L under the control condition in the WT (Figure 4b, lane 1), suggesting that Herp, Derlin-2, HRD1, and SEL1L were present in the same complex. This inference was further confirmed by sequential coimmunoprecipitation experiments (Figure 5). In the first coimmunoprecipitation, Herp and its binding proteins were coimmunoprecipitated with anti-Herp antibodies and eluted with an immunogen peptide of Herp. The resultant eluate was subjected to a second coimmunoprecipitation with anti-Derlin-2 antibodies, and the captured proteins were analyzed using Western blotting. HRD1 was detected in the second eluate (Figure 5, lane 3), indicating that Herp, Derlin-2, and HRD1 were simultaneously present in the same complex (Complex-1a) under the control condition in the WT liver.

Under the ER stress conditions, the interactions among Herp, Derlin-2, HRD1, and SEl1L were dramatically altered, which was confirmed using Western blotting. The amount of Derlin-2 coimmunoprecipitated with anti-Herp antibodies was drastically reduced following Tm treatment (Figure 4b, lanes 1 and 2), indicating that the interaction between Herp and Derlin-2 was attenuated by ER stress. Noticeably, Derlin-3 joined the complex containing Herp, HRD1, and nonglycosylated SEL1L under the ER stress conditions (Figure 4b, lane 2), suggesting that Derlin-2 in Complex-1a was replaced by Derlin-3. This resulted in the formation of another complex (Complex-1b) containing Herp, Derlin-3, and HRD1. The interaction between Herp and HRD1 showed no obvious change under ER stress (Figure 4b, lanes 1 and 2). The observation was also confirmed by the coimmunoprecipitation experiments using anti-HRD1 antibodies (Supplemental Figure S1a,d).

### 2.6. Physical Interactions among ERAD Factors—Complex-2

We also performed coimmunoprecipitation experiments using anti-Derlin-1 antibodies (Figure 4b, right panels). Under the control condition, Derlin-1 was a part of the complex containing Derlin-2 and the binding between Derlin-1 and Derlin-2 was enhanced under ER stress (Figure 4b, lanes 7 and 8). The other ERAD factors, VIMP and p97, were also coimmunoprecipitated with Derlin-1 in an ER stress-dependent manner. These interactions were confirmed by coimmunoprecipitation experiments using anti-Derlin-2 antibodies (Supplemental Figure S1f), suggesting the presence of a complex (Complex-2) containing Derlin-1, Derlin-2, VIMP, and p97.

The existence of the complex containing Derlin-1 was further confirmed by sequential coimmunoprecipitation experiments (Figure 5, right panels). In the first coimmunoprecipitation, Derlin-1 and its binding proteins were trapped with anti-Derlin-1 antibodies and eluted with an immunogen peptide of Derlin-1. The resultant eluate was subjected to a second coimmunoprecipitation with anti-Derlin-2 antibodies, and the captured proteins were analyzed using Western blotting. VIMP and p97 were coimmunoprecipitated, suggesting that they were present in the same complex. In this experimental condition, Herp and Derlin-3 were not detected in the second eluates, indicating that Derlin-1 and Herp/Derlin-3 exist in different complexes.

Of note, anti-Herp antibodies did not coimmunoprecipitate Derlin-1, whereas anti-Derlin-1 antibodies did coimmunoprecipitate Herp (Figure 4b). This discrepancy may be explained by the binding stoichiometry of Herp and Derlin-1: Derlin-1 was bound to a small part of the Herp molecules, and Herp was bound to most of the Derlin-1 molecules. We assume that there is an interaction between the two complexes, Complex-1b and Complex-2. Therefore, our data are consistent with the findings of previous reports demonstrating the presence of a complex containing Herp, Derlin-1, and HRD1 [17,18,19].

### 2.7. Roles of Herp and Derlin-3 on the Physical Interactions among ERAD Factors

In our previous study, we generated HKO mice and demonstrated the deleterious effect of Herp deficiency on the degradation of ERAD substrates in the liver [29]. In the present study, we found that the accumulation of the nonglycosylated forms of SEL1L and calnexin in HKO liver was more remarkable than in WT (Figure 4a, lanes 2 and 6). This suggested that Herp deficiency led to a dysfunction of the ERAD factors. Although the ER stress-induced interactions among Derlin-1, Derlin-2, Derlin-3, VIMP, and p97 were observed not only in WT but, also, in HKO livers (Figure 4b, lanes 8 and 12), the interaction between HRD1 and Derlin-2 was significantly reduced in the HKO liver under the control condition (Supplemental Figure S1a). These results suggested that Herp was important for the interaction between Derlin-2 and HRD1 but dispensable for the interactions among Derlin-1, Derlin-2, Derlin-3, VIMP, and p97.

We next investigated the effects of Derlin-3 deficiency on each ERAD factor and their complexes. The accumulation of the nonglycosylated forms of SEL1L and calnexin in the 3KO liver was more remarkable than in the WT (Figure 4a, lanes 2 and 4), suggesting that Derlin-3 deficiency also led to a dysfunction of ERAD factors. It should be noted that the ER stress-dependent decrease in the interaction between Herp and Derlin-2 was drastically inhibited in 3KO (Figure 4b, lanes 1–4) and that the increase in the binding of Derlin-1 and Derlin-2 following ER stress was apparently suppressed in 3KO (Figure 4b, lanes 7–10). These findings suggested that Derlin-3 replaced Derlin-2 as the binding partner of Herp, and Derlin-2 released from Herp interacted with Derlin-1 under ER stress. In the 3KO liver, the binding of Derlin-1 and Herp under ER stress was also significantly suppressed (Figure 4b, lanes 7–10), which will be discussed later in Section 2.11. Thus, Derlin-3 seems to play an important role in the regulation of the ERAD complex composition in response to ER stress.

### 2.8. ER Stress Changes the Distribution of ERAD Factors in Density Gradient Fractions

ERAD factors were reported to exhibit bimodality when fractionated by sucrose density gradient centrifugation [40,41]. We confirmed the same in the present study using mouse liver samples, and we further observed the changes in the distribution of ERAD factors induced by ER stress. In the WT mice liver, Herp, Derlin-1, Derlin-2, and HRD1 were widely distributed with two peaks: one in the light fractions (Supplemental Figure S2a, upper panels, fractions 8–12) and the other in the heavy fractions (fractions 18–22). As expected from the coimmunoprecipitation experiments described above, the distributions of these proteins were altered under ER stress (Supplemental Figure S2a, lower panels) as follows. The quantitative balance of Herp, Derlin-1, Derlin-2, and HRD1 was shifted toward the heavy fractions (Supplemental Figure S2b). However, Derlin-3 was detected throughout all fractions (Supplemental Figure S2a, lower panels) in the Tm-treated liver, suggesting that Derlin-3 was newly expressed and incorporated into the ERAD complexes under ER stress. These findings suggest that the composition of ERAD complexes are dynamically regulated as per the ER state. Furthermore, the imperfect match in the distributions of ERAD factors suggested that these proteins may not be present in the same protein complex.

### 2.9. ERAD Complexes in the Light and Heavy Fractions

**Figure 6:**
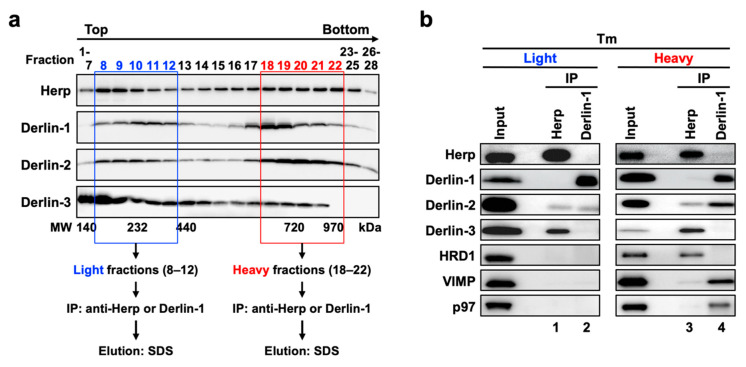
Physical interactions among ERAD factors in the light and heavy fractions obtained from sucrose density gradient centrifugation. (**a**) Experimental workflow. Liver microsomes prepared from Tm-treated WT mice were subjected to sucrose density gradient centrifugation, and the light (8–12) and heavy (18–22) fractions were used for the coimmunoprecipitation experiments. (**b**) Coimmunoprecipitation analyses on the light and heavy fractions. The eluates were analyzed by Western blotting using antibodies indicated on the left side. IP, immunoprecipitation.

To investigate the correlation among the ERAD complexes (Complex-1a, Complex-1b, and Complex-2) in the bimodal distribution of the proteins in sucrose density gradient fractions, we performed coimmunoprecipitation experiments using the collected fractions. The fractions 8–12 and 18–22 under the ER stress conditions were used as the light and heavy fractions, respectively (Figure 6a). Anti-Herp antibodies coimmunoprecipitated Derlin-3 and a small portion of Derlin-2 in the light fractions (Figure 6b, lane 1). The same antibodies coimmunoprecipitated HRD1 in addition to Derlin-3 and a small portion of Derlin-2 in the heavy fractions (lane 3). These results suggested that the participation of HRD1 completes the formation of Complex-1b in the heavy fractions, a portion of which interacts with Derlin-2. Complex-1b also seemed to interact with a small portion of VIMP and p97 (lane 3).

The interaction between Derlin-1 and Derlin-2 appeared much stronger in the heavy fractions (Figure 6b, lane 4) than in the light fractions (lane 2). As is the case in the sequential coimmunoprecipitation (Figure 5), the interaction between Derlin-1 and Herp or Derlin-3 was not observed. In addition, VIMP and p97 were unambiguously coimmunoprecipitated with Derlin-1 in the heavy fractions (Figure 6b, lane 4) but not in the light fractions (lane 2). These results suggested that the complex containing Derlin-1, Derlin-2, VIMP, and p97 (Complex-2) formed a high-molecular-weight complex in the heavy fractions. It was consistent with our observation that p97 and VIMP were absent in the complexes containing Herp (Complex-1a and Complex-1b) but present in the complex containing Derlin-1 (Complex-2) (Figure 5).

The apparent molecular masses of the protein complexes in the light and heavy fractions were approximately 200–400 kDa and >500 kDa, respectively, as estimated from the molecular weight marker proteins (Supplemental Figure S2a). Thus, the molecular sizes of these complexes were larger than the sum of the masses of the individual ERAD factors that they contained. Other ERAD factors such as SEL1L, EDEM, and OS-9 may also colocalize with the complexes, and Derlin-1 and HRD1 can each form homo-oligomers [23,42]. When we co-expressed FLAG-tagged Herp and HA-tagged Herp, these proteins were reciprocally coimmunoprecipitated (data not shown). Thus, these complexes may contain not only the hetero-oligomers but, also, homo-oligomers of ERAD factors. In addition, these complexes may contain ERAD substrates to be degraded. Further protein analyses are needed in order to elucidate the full contents of the ERAD complexes and the functional differences among Complex-1a, Complex-1b, and Complex-2.

### 2.10. Role of Derlin-3 in the Formation of ERAD Complexes

**Figure 7:**
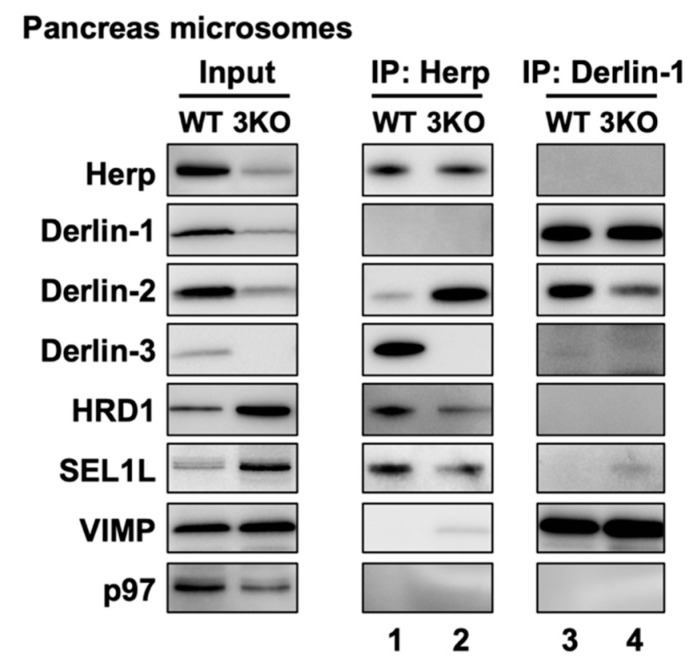
Effects of Derlin-3 deficiency on the mouse pancreas ERAD complex formation. WT and 3KO mice were fasted for 12 h before euthanasia. Pancreas were homogenized and subjected to subcellular fractionation using centrifugation. Digitonin-solubilized pancreas microsomes were prepared, and coimmunoprecipitation was performed using Herp and Derlin-1 antibodies. The input and eluates were analyzed by Western blotting using antibodies indicated on the left side.

In the liver, Derlin-3 is important for ERAD complex changes in response to ER stress (Figure 4). Then, we investigated the role of Derlin-3 in the pancreas, which is considered to be in a chronic ER stress state. Unlike the liver, Derlin-3 is constitutively expressed in the pancreas (Figure 1, Figure 2 and Figure 3) and is required for the regulation of the expression levels of ERAD factors (Figure 1, Figure 2 and Figure 3). In the WT pancreas under normal conditions, Derlin-3 was efficiently coimmunoprecipitated with anti-Herp antibodies, while Derlin-1 and Derlin-2 were not (Figure 7, lane 1). On the contrary, Derlin-2 was efficiently coimmunoprecipitated with anti-Derlin-1 antibodies, while Derlin-3 and Herp were not (lane 3). These results suggested that Complex-1b and Complex-2 were present in the WT pancreas, similar to those in the liver under ER stress (Figure 4). This observation is in good agreement with the chronic ER stress conditions of the pancreas.

Next, we compared the ERAD complexes between the WT and 3KO pancreas under the normal (i.e., chronic ER stress) conditions. In the 3KO pancreas, the binding of Herp and Derlin-2 was markedly increased as compared to that in the WT pancreas (Figure 7, lanes 1 and 2). In contrast, the binding of Derlin-1 and Derlin-2 was decreased (lanes 3, and 4). These are strikingly similar to the differences in the presence or absence of ER stress in the liver (Figure 4b, lanes 2, 4, 8 and 10). Thus, both in the liver and in the pancreas, Derlin-3 may have a similar role to determine whether Derlin-2 binds to Herp or Derlin-1. In this context, the presence or absence of Derlin-3 would be a key factor for the formation of ERAD complexes containing Herp and Derlins.

### 2.11. Dynamic Nature of ERAD Complexes in Response to ER Stress

**Figure 8:**
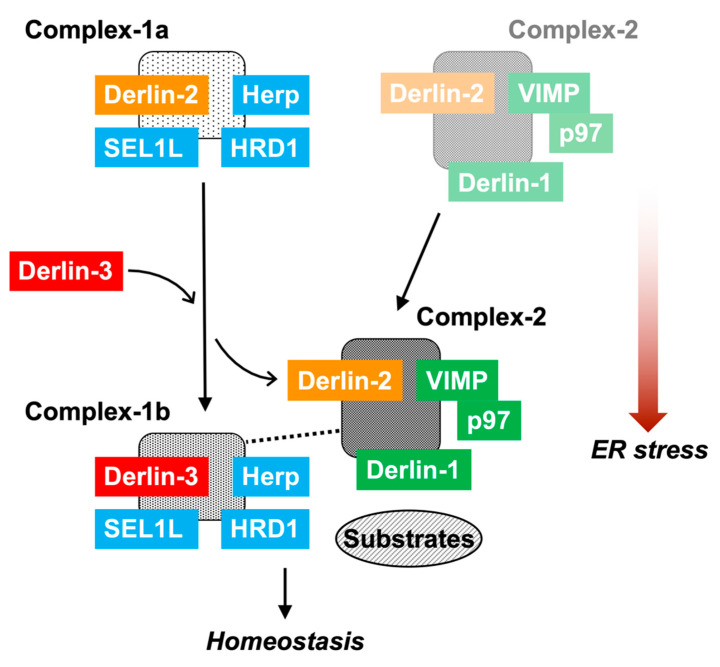
The role of Derlin-3 in ERAD complex formation. In the non-ER stress conditions, there are Complex-1a containing Herp, Derlin-2, HRD1, and SEL1L and a small amount of Complex-2 containing Derlin-1, Derlin-2, VIMP, and p97 on the ER membrane. In the liver, ER stress induces the expression of Derlin-3. Then, Derlin-3 is replaced by Derlin-2 to form Complex-1b containing Herp, HRD1, SEL1L, and Derlin-3. Subsequently, Derlin-2 upregulates the formation of Complex-2. Deficiency of Herp suppresses the formation of Complex-1a. Deficiency of Derlin-3 suppresses the change from Complex-1a to Complex-1b, and simultaneously, the interaction between Complex-1b and Complex-2 disappears. In the pancreas, there is chronic ER stress; therefore, Complex-1b and Complex-2 remain stationary. Deficiency of Derlin-3 results in the formation of Complex-1a and a decrease in the formation of Complex-1b and Complex-2.

The data in the present study demonstrated the multiple and variable types of ERAD complexes in the ER membrane (Figure 8). The data also shows that Derlin-3 has an important role in the formation of the ERAD complex in response to ER stress. In this section, we propose a model describing the dynamic nature of ERAD complexes in response to ER stress. Complex-1a contains Herp, Derlin-2, HRD1, and SEL1L under normal conditions. Under ER stress, Derlin-2 in Complex-1a is substituted by newly expressed Derlin-3, resulting in the formation of Complex-1b containing Herp, Derlin-3, HRD1, and SEL1L. Derlin-3 deficiency disturbs the dissociation of Derlin-2 from Complex-1a under ER stress (Figure 4). This suggests a phase-switching function of Derlin-3 in response to ER stress. Complex-2, upregulated in the ER stress conditions, contains Derlin-1, Derlin-2, VIMP, and p97. Derlin-2 translocates from Complex-1a to Complex-2 in response to ER stress (i.e., by the presence of Derlin-3). The formation of Complex-1a, particularly the interaction between HRD1 and Derlin-2, is affected due to the absence of Herp; however, the formation of Complex-2 is not affected. Under ER stress, the interaction between Herp and Derlin-1 was observed (Figure 4), suggesting a physical interaction between Complex-1b and Complex-2 under ER stress. These observations are consistent with the previous studies on the interactions among ERAD factors [17,18,19].

The interaction between Complex-1b and Complex-2 was not observed in the absence of Derlin-3. Thus, Derlin-3 probably functions as a hub molecule to control the binding of ERAD factors with each other. The function of Derlin-3 is not compensated by the other family members, Derlin-1 and Derlin-2. In the Derlin-3-deficient pancreas, ERAD substrate proteins such as HRD1 and nonglycosylated forms of SEL1L and calnexin were accumulated. It is unclear how the dynamic changes in the ERAD complexes alter ERAD activity and coping with ER stress. Further analysis is needed to address the functional differences among Complex-1a, Complex-1b, and Complex-2. Derlin-3 has been reported to be involved in pathological conditions such as ischemic heart, colon cancer, breast cancer, and gastric cancer [30,31,32,33]. These phenotypes may also be caused by an aberrant composition of ERAD complexes.

A limitation of the present study is that we biochemically analyzed ERAD complexes of two representative organs (pancreas and liver) that express Derlin-3 or not under normal conditions. Other organs and cells may have different ERAD complexes. Another limitation is that we did not investigate all ERAD factors because of antibody unavailability. Proteomic analyses of purified ERAD complexes may bring new insights into the ERAD system. Despite these limitations, our findings succeeded in revealing a novel role of Derlin-3 in the ERAD system. It will be interesting and important to study how the abnormal function of ERAD complexes caused by Derlin-3 deficiency exerts an influence on physiological homeostasis.

## 3. Materials and Methods

### 3.1. Ethics Statement

All live mouse experiments were approved by the Animal Care and Use Committee of the National Cerebral and Cardiovascular Center (approval code 15016, 18 March 2015) and were performed in accordance with institutional and national guidelines and regulations. All dissections were performed after euthanasia, and all efforts were made to minimize suffering.

### 3.2. Western Blotting

Anti-Herp and anti-Derlin-3 antibodies were raised by inoculating rabbits with the keyhole limpet hemocyanin-conjugated peptides SDGLRQREVLRNLS (residues 122–135 of mouse Herp) and DPDYLPLPEEQPEL (residues 215–228 of mouse Derlin-3) [29]. Anti-Derlin-1 (MBL, Nagoya, Japan), anti-Derlin-2 (MBL) and anti-HRD1 (Abgent, San Diego, CA, USA), anti-SEL1L (Alexis Biochemicals, San Diego, CA, USA), anti-VIMP (Sigma-Aldrich, Darmstadt, Germany), anti-p97 (Santa Cruz Biotechnology, Dallas, TX, USA), anti-Calnexin (Stressgen Biotechnologies, San Diego, CA, USA), and normal rabbit IgG (MBL) were purchased from the indicated suppliers.

Protein samples were subjected to SDS-PAGE and transferred to polyvinylidene difluoride membranes (Bio-Rad Laboratories, Hercules, CA, USA). Membranes were blocked with 5% skim milk, incubated with primary antibodies, and then incubated with peroxidase-labeled anti-rabbit or mouse IgG (Kirkegaard & Perry Laboratories, Gaithersburg, MD, USA). Signals were detected by chemiluminescence using Immobilon Western (Merck Millipore, Burlington, VT, USA) and LAS-3000 (Fujifilm, Tokyo, Japan).

### 3.3. Tissue Preparation

To validate the tissue expression profiles of the ERAD factors, 12 h-fasted male WT and 3KO mice were euthanized, and their organs were excised. The organs were homogenized in a SDS-PAGE sample buffer using electric homogenizer T10 basic (IKA, Staufen, Germany). The total lysates were subjected to SDS-PAGE, followed by Western blotting using the indicated antibodies. Equal loading of the samples was confirmed by protein quantitation in gels stained with GelCode Blue (Thermo Fisher Scientific, Waltham, MA, USA).

To investigate the stabilization of the protein levels in mice pancreas and livers, the pancreas or liver lysates were prepared from WT and 3KO mice. The lysates were homogenized in PBS and then incubated on ice for the indicated time points. The lysates were immediately processed in the SDS-PAGE sample buffer and subjected to Western blotting.

To examine the protein levels of the ERAD factors in the pancreas and liver, fasted male WT, 3KO, HKO, and 3HKO mice were euthanized, and their livers and pancreas were excised. The preparation of homogenates and Western blotting were performed as described above.

To investigate the effects of ER stress, fasted male WT, 3KO, and HKO mice were intraperitoneally injected with PBS or Tm (2-µg/g body weight) to induce ER stress caused by inhibiting glycosylation. Twenty-four hours after injection, the mice were euthanized, and their livers were excised. The livers were homogenized in isotonic buffer (10-mM HEPES, 220-mM mannitol, and 70-mM sucrose; pH 7.4) containing protease inhibitor cocktail tablets (cOmplete, Roche, Basel, Switzerland) and subjected to subcellular fractionation as described below.

### 3.4. Subcellular Fractionation

The liver or pancreas lysates were centrifuged at 1100× *g* for 10 min, and the resultant supernatants were centrifuged at 8300× *g* for 10 min. The clarified supernatants were then centrifuged at 100,000× *g* for 1 h to precipitate microsomes. The pellets were solubilized on ice for 30 min in 1% digitonin, and the debris was removed by centrifugation at 100,000× *g* for 20 min. The supernatants (solubilized microsomes) were used for coimmunoprecipitation experiments (Figure 4, Figure 5, and Figure 7 and Supplemental Figure S1) and sucrose density gradient centrifugation experiments (Figure 6 and Supplemental Figure S2).

### 3.5. Coimmunoprecipitation

The liver or pancreas microsomes were lysed on ice for 30 min in lysis buffer (50-mM Tris-HCl, 150-mM NaCl, and 1% digitonin; pH 7.5) containing protease inhibitor cocktail tablets (cOmplete). Undissolved residues were cleared by centrifugation at 100,000× *g* for 20 min, and the supernatants were subjected to coimmunoprecipitation as described below. The light and heavy fractions from sucrose density gradient centrifugation were used directly for coimmunoprecipitation experiments.

Antibodies against Herp, Derlin-1, Derlin-2, and HRD1 or normal IgG were added to the samples and incubated for 30 min at 4 °C. Subsequently, the samples were incubated with Protein A-Sepharose beads (GE Healthcare, Chicago, IL, USA) for 30 min at 4 °C. After washing with the lysis buffer, proteins bound to the beads were eluted in the SDS-PAGE sample buffer (Figure 4, Figure 6, and Figure 7 and Supplemental Figure S1) or in the lysis buffer containing 200-µg/mL immunogen peptide (SDGLRQREVLRNLS for Herp and RHNWGQGFRLGDQ for Derlin-1) for the sequential coimmunoprecipitation experiments (Figure 5). For the second phase of the sequential coimmunoprecipitation experiments, anti-Derlin-2 antibodies were added to the eluates from the first coimmunoprecipitation and incubated for 30 min at 4 °C. Further, the samples were incubated with Protein A-Sepharose beads. Proteins on the beads were eluted in the SDS-PAGE sample buffer and analyzed using Western blotting. Coimmunoprecipitation with normal IgG was also performed at each step to monitor the nonspecific precipitation (e.g., Supplemental Figure S1g).

### 3.6. Sucrose Density Gradient Centrifugation

The liver supernatants were loaded onto a 0.15–1-M sucrose linear gradient and centrifuged at 284,000× *g* for 6 h at 4 °C. The fractions were serially collected from the bottom of the centrifuge tubes (0.45 mL/fraction, 28 fractions/tube). The fractions were subjected to SDS-PAGE, followed by Western blotting (Supplemental Figure S2a). Alternatively, for coimmunoprecipitation experiments, fractions 8–12 and 18–22 (Figure 6) were pooled as the light and heavy fractions, respectively. Molecular-weight marker proteins (bovine serum albumin, lactate dehydrogenase, catalase, ferritin, and thyroglobulin) were also separated by sucrose density gradient centrifugation under the same conditions.

## Supplementary Materials

The following are available online at https://www.mdpi.com/1422-0067/21/17/6146/s1: Figure S1: Coimmunoprecipitation using the mouse liver with or without ER stress. Figure S2: Distribution of ERAD factors in sucrose density gradients.

## Abbreviations

ER	Endoplasmic reticulum
ERAD	ER-associated protein degradation
WT	Wild-type
3KO	Derlin-3-deficient
iBAT	Interscapular brown adipose tissue
PBS	Phosphate-buffered saline
3HKO	Derlin-3/Herp double-deficient
HKO	Herp-deficient
Tm	Tunicamycin
C	Control
IP	Immunoprecipitation
