# Supplementary material for: Derlin-3 Is Required for Changes in ERAD Complex Formation under ER Stress
Source: Int J Mol Sci. 2020 Aug 26;21(17):6146. doi: 10.3390/ijms21176146 (PMC7504720; doi:10.3390/ijms21176146)
Supplement: Supplementary file 1 [file ijms-21-06146-s001.pdf]

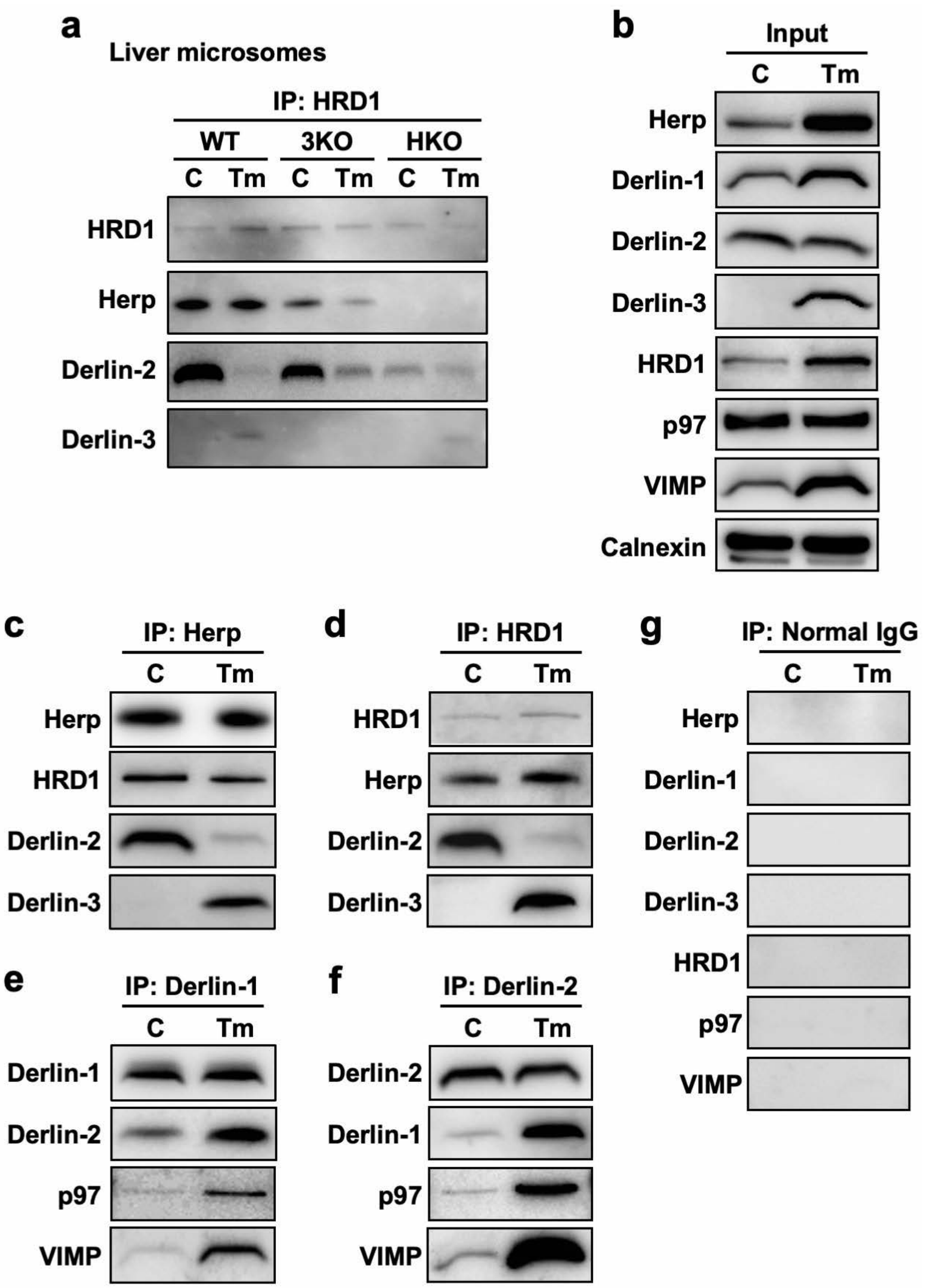

Supplementary Figure S1. Coimmunoprecipitation using the mouse liver with or without ER stress. (**a**) Digitonin-solubilized liver microsomes obtained from indicated genotypes of mice were prepared as shown in Figure 4, and coimmunoprecipitation was performed using HRD1 antibodies. The input and eluates were analyzed using western blotting using antibodies indicated on the left side. The loading volumes of samples were adjusted to equalize the band intensities of the directly immunoprecipitated HRD1 among different conditions. (**b**–**g**) Digitonin-solubilized liver microsomes of WT were prepared as shown in Figure 4, and coimmunoprecipitation was performed using indicated antibodies. The input (**b**) and eluates (**c**–**g**) were analyzed using western blotting using antibodies indicated on the left side. The loading volumes of samples were adjusted to equalize the band intensities of the directly immunoprecipitated target proteins among different conditions. The presented data were obtained from two independent experiments. IP, immunoprecipitation.

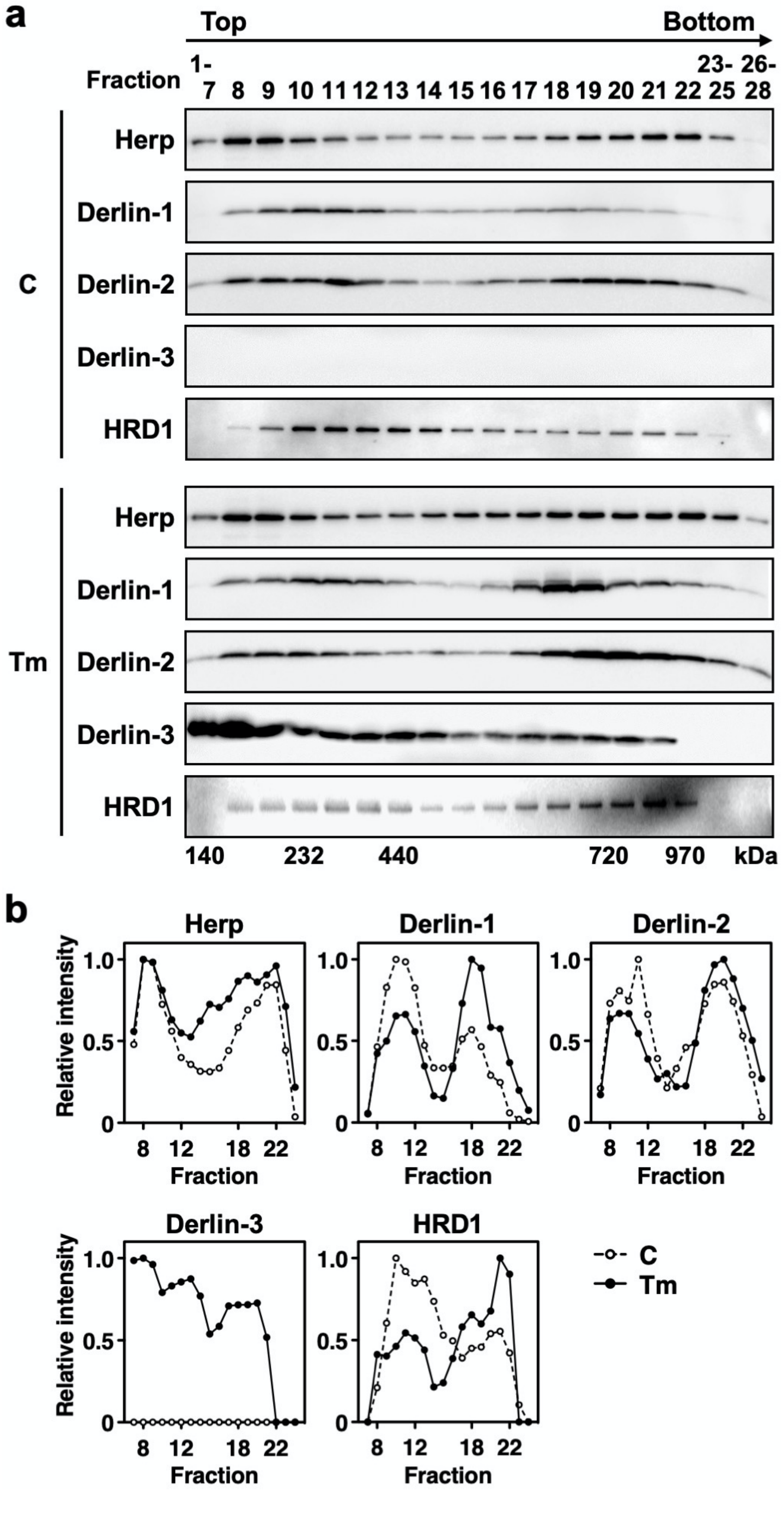

Supplementary Figure S2. Distribution of ERAD factors in sucrose density gradients. (**a**) Liver microsomes were prepared from WT mice that had been injected intraperitoneally with PBS (C) or Tm 24 h before euthanasia. Digitonin-solubilized microsomes were subjected to sucrose density gradient centrifugation, and the collected fractions were analyzed using western blotting using the indicated antibodies. (**b**) The band intensities were relatively quantitated using Image Gauge (Fujifilm) and plotted. The maximum value of each protein intensity was defined as 1.0. The presented data were obtained from two independent experiments.
